# The Role of Collagen Triple Helix Repeat-Containing 1 Protein (CTHRC1) in Rheumatoid Arthritis

**DOI:** 10.3390/ijms22052426

**Published:** 2021-02-28

**Authors:** Askhat Myngbay, Limara Manarbek, Steve Ludbrook, Jeannette Kunz

**Affiliations:** 1 PhD Program in Science Engineering and Technology, Nazarbayev University, Nur-Sultan 010000, Kazakhstan; askhat.myngbay@nu.edu.kz; 2 Department of Biology, School of Sciences and Humanities, Nazarbayev University, Nur-Sultan 010000, Kazakhstan; lmanarbek@nu.edu.kz; 3 GlaxoSmithKline Research & Development, Stevenage SG1 2NY, UK; Steve.B.Ludbrook@gsk.com

**Keywords:** collagen triple helix repeat containing 1, CTHRC1, rheumatoid arthritis, biomarker, bone erosion, cartilage destruction, fibroblast-like synoviocytes, Wnt signaling

## Abstract

Rheumatoid arthritis (RA) is a chronic autoimmune disease causing inflammation of joints, cartilage destruction and bone erosion. Biomarkers and new drug targets are actively sought and progressed to improve available options for patient treatment. The Collagen Triple Helix Repeat Containing 1 protein (CTHRC1) may have an important role as a biomarker for rheumatoid arthritis, as CTHRC1 protein concentration is significantly elevated in the peripheral blood of rheumatoid arthritis patients compared to osteoarthritis (OA) patients and healthy individuals. CTHRC1 is a secreted glycoprotein that promotes cell migration and has been implicated in arterial tissue-repair processes. Furthermore, high *CTHRC1* expression is observed in many types of cancer and is associated with cancer metastasis to the bone and poor patient prognosis. However, the function of CTHRC1 in RA is still largely undefined. The aim of this review is to summarize recent findings on the role of CTHRC1 as a potential biomarker and pathogenic driver of RA progression. We will discuss emerging evidence linking CTHRC1 to the pathogenic behavior of fibroblast-like synoviocytes and to cartilage and bone erosion through modulation of the balance between bone resorption and repair.

## 1. Introduction

Rheumatoid arthritis (RA) is a chronic systemic autoimmune disease, which affects around 1% of the population according to the World Health Organization [1]. RA is characterized by cartilage degradation and bone erosion within both small and larger joints including hand, wrist, knee and feet, leading to disability in a proportion of patients [2]. Although several genetic and environmental factors have been linked to an increased risk for RA, the definitive pathogenesis remains obscure, making the development of effective treatment strategies challenging. Some breakthroughs, including the introduction of anti-tumor necrosis factor alpha (anti-TNF-α), in the treatment of RA, which occurred in the mid-1990s, showed efficacy towards inflammation and joint destruction and led to an improvement of clinical outcomes of RA [3]. However, cytokine antagonists against TNF-α, Interleukin-6 (IL-6) and Interleukin-1 (IL-1) lacked efficacy in a significant fraction of patients, and the persistence of efficacy remains a tremendous problem even in responsive patients [3]. Likewise, the therapeutic effects of B cell depletion and T cell co-stimulation blockers were not observed in all patients [4,5]. Indeed, accumulating evidence suggests that differences in genetic background and exposure to environmental stimuli among patients require treatment strategies to be more personalized as particular patients’ response can be TNF-α-dominant, T cell-dominant, and B cell-dominant [4,5].

While significant efforts have been undertaken to identify biomarkers to diagnose RA, there is still a lack of diagnostic and prognostic biomarkers for better patient stratification. ACPA (anti-citrullinated protein antibody) and RF (rheumatoid factor) are two widely accepted autoantibodies used in clinical practice as biologic markers of RA [6]. Nonetheless, neither of these two markers has sufficient specificity or sensitivity for effective diagnosis of all RA patients, and neither autoantibody allows classification of patient subpopulations or patient outcome [6]. Recently, CTHRC1 has emerged as a new biomarker that may contribute to improved RA diagnosis and assessment of disease activity [7,8]. CTHRC1 protein levels are increased in the plasma of RA patients but were either absent or detected only at very low levels in healthy individuals or patients suffering from other forms of arthritis, such as OA or reactive arthritis (ReA) [7]. These findings suggest that CTHRC1 may enhance differential diagnosis of RA when used in a wider panel of markers. In addition, emerging evidence suggests that CTHRC1 is directly involved in the disease course. Accordingly, CTHRC1 is expressed in subsets of activated fibroblast-like (FLS) cells of the synovium associated with RA pathophysiology [9]. Furthermore, CTHRC1 acts as a critical modulator of bone resorption and formation by regulating osteoclast–osteoblast crosstalk [10,11], thus raising the possibility that CTHRC1 levels may reflect a more direct role—pathological or protective—in cartilage and bone erosion in RA. In this review, we will discuss the role and diagnostic potential of CTHRC1 in RA and provide an overview of the signaling processes modulated by CTHRC1.

## 2. CTHRC1 Domain Structure

**Figure 1:**
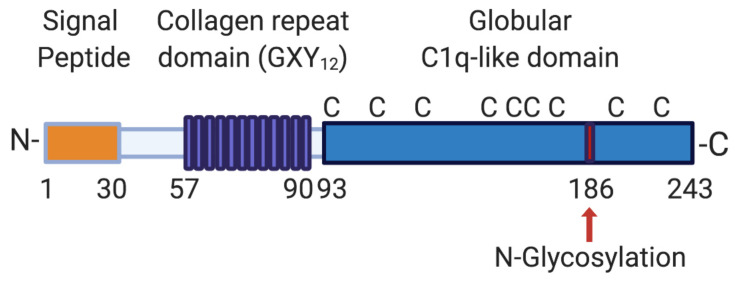
Domain structure of human CTHRC1. The longest CTHRC1 variant encodes a 243 amino acid protein with a 30 amino acid cleavable signal sequence, followed by a short collagen repeat domain consisting of 12 GXY repeats (where G stands for glycine, X for any amino acid and Y for tyrosine). CTHRC1 also contains a globular cysteine-rich (C indicates the positions of individual cysteine residues) domain with structural similarity to C1q. CTHRC1 is glycosylated, and a single potential glycosylation site is indicated with a red arrow. Aminoterminal (N-) and carboxyterminal (-C) ends of the protein are indicated. Numbers specify amino acid positions within the human protein. Created with BioRender.com.

CTHRC1 is a secreted 28 kDa glycoprotein that is highly conserved from chordates to vertebrates [12]. The human protein contains an N-terminal hydrophobic signal peptide of 30 amino acids in length that directs CTHRC1 for secretion, a short collagen triple helix repeat (CTHR) domain consisting of 12 repeats of the Gly-X-Y motif located between amino acids 57 and 93 [12], and a highly conserved C-terminal domain with structural homology to the globular C1q domain of collagens VIII and X domain (Figure 1) [13]. The CTHR domain may promote CTHRC1 dimer or trimer formation and mediate interaction with a variety of ligands [12,13]. The domain structure of CTHRC1 is similar to that of adiponectin, which belongs to the C1q/tumor necrosis factor (TNF)-related protein superfamily [14]. Thus, secreted CTHRC1 may share structural characteristics with this protein superfamily and similarly assemble into trimers and multimers composed of CTHRC1 trimers. CTHRC1 is also a cysteine-rich protein: 10 conserved cysteine residues are distributed between residues 55 to 243 (9 of which are located within the C1q domain; Figure 1) and account for 4.7% of amino acid residues present in the mature protein [12]. The single putative N-glycosylation site at amino acid 186 (Figure 1) has been reported to stabilize the protein and may promote the tethering of secreted CTHRC1 to the plasma membrane [15]. Several shorter alternatively spliced transcripts have been described [12], one of which lacks the N-terminal hydrophobic signal peptide and may not be secreted. However, only the longest transcript has so far been functionally characterized in more detail.

## 3. Identification and Physiological Function of CTHRC1

CTHRC1 was first identified by Pyagay et al., who reported the transiently upregulated expression of *CTHRC1* in rat arteries on adventitial and intimal smooth muscle following injury [12]. Increased levels of CTHRC1 protein were also observed in the matrix of calcifying human atherosclerotic plaques [12]. Notably, CTHRC1 was not detectable in normal arteries, indicating that the protein plays a specific role in the wound-healing response and promotes vascular remodeling during arterial injury [12,16]. Mechanistically, increased CTHRC1 levels are associated with a significant decrease in collagen type I and type III mRNA and protein levels, leading to a reduction of collagen deposition, and enhanced migration [12]. Consistent with such a role, CTHRC1 expression has been correlated with conditions and processes associated with deregulated wound and tissue repair, including, among others, liver and lung fibrosis and myocardial infarction [17,18,19,20,21,22,23,24], liver injury caused by Hepatitis B infection [20,25,26], tumor angiogenesis and metastasis [27,28].

## 4. Expression of *CTHRC1*

Under non-pathological conditions, *CTHRC1* expression is detected mainly during embryonic development in the visceral endoderm, the notochord and neural tube and is also significantly associated with developing cartilage and bone, especially in calcified tissues [29]. In the adult body, expression of *CTHRC1* is much more restricted and observed mainly in the bone matrix and periosteum, in the myocardium and in renal arteries [29]. In general, *CTHRC1* expression is detected and increased specifically in stromal cell types, including myofibroblasts and smooth muscle cells [29,30]. In addition, *CTHRC1* is expressed in human pituitary glands and was proposed to act as a circulatory hormone [31]. Overall, the restricted expression in the adult body likely accounts for the low levels of CTHRC1 protein normally detected in circulation.

## 5. Signaling Roles of CTHRC1

The pathways modulated by CTHRC1 reflect its role in tissue remodeling after injury, regulation of ossification and other physiological processes, most significantly cancer development and progression to metastasis. Extensive evidence links CTHRC1 to two major signaling pathways: The transforming growth factor β (TGF-β) pathway and canonical/noncanonical Wnt signaling pathways.

### 5.1. Role of CTHRC1 in the TGF-β Pathway

The TGF-β superfamily of signaling pathways regulates diverse developmental and homeostatic processes and alterations in these pathways are associated with a variety of human pathologies, including developmental disorders, vascular and immune diseases, fibrosis, and cancer [32,33,34]. The relationship between CTHRC1 with members of the TGF-β superfamily is highlighted by their overlap in expression patterns in specific cell types and tissues [10,16] and the finding that the promoter region of CTHRC1 has a consensus binding site for SMAD transcription factors, which are downstream components of TGF-β/BMP4 pathways [35]. Consistent with this, CTHRC1 expression was shown to be induced by TGF-β and TGF-β superfamily members, including Bone morphogenetic protein 2 and 4 (BMP2/4) and activin [10,16,36].

**Figure 2:**
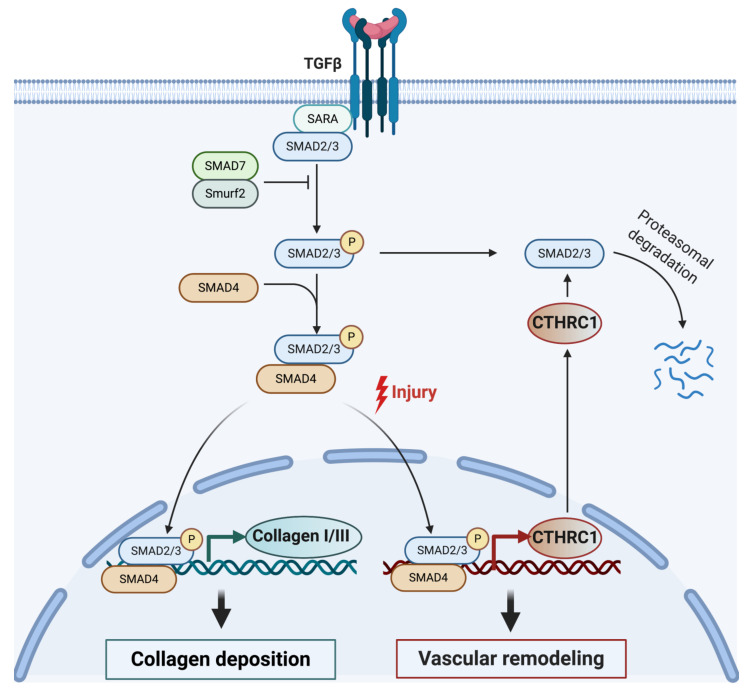
Model for CTHRC1 modulating collagen deposition and vascular repair following injury through the TGF-β signaling pathway. TGF-β is known to stimulate collagen deposition in various cell types by activating receptor-associated SMAD proteins (SMAD2 and SMAD3). Activated SMAD2 and SMAD3 oligomerize with SMAD4. The complex then translocates into the nucleus, where it regulates the expression of TGF-β-responsive genes, including genes encoding collagen I and III, and CTHRC1. Elevated levels of CTHRC1 eventually lead to attenuation of TGF-β signaling by inhibiting SMAD2/3 phosphorylation [36], possibly leading to SMAD2/3 degradation [21]. Created with BioRender.com.

The link between TGF-β and CTHRC1 was first reported in injured arteries and has since also been reported in other cell types [11,17,19,21,22,29]. TGF-β is a central growth factor involved in vascular development in the embryo and the wound healing response in adult tissues [12,16,36]. During vascular development and upon injury, TGF-β mediates several negative regulatory effects during vessel repair by upregulating collagen synthesis via activation of SMAD2/3 complexes, leading to increased collagen deposition and smooth muscle cell proliferation [16]. Activation of the TGF-β signaling pathway also induces expression of *CTHRC1* [36]. Interestingly, CTHRC1 has been shown to act as an inhibitor of TGF-β, impacting neointimal formation and proliferation and migration of smooth muscle cells after vascular injury [10,16,36]. Accordingly, sustained activation of *CTHRC1* expression confers an antagonistic effect on TGF-β signaling with CTHRC1, leading to reduced SMAD2/3 phosphorylation in vascular cells (Figure 2) [36]. Elevated levels of CTHRC1 thereby promote vessel repair by inhibiting the expression of the TGF-β target genes collagen type I and III, which, in turn, reduces collagen deposition and enhances cell migration during vascular remodeling [12,16,36]. This regulatory feedback loop (summarized in Figure 2) may allow for tight control of CTHRC1 activity to balance the effects of TGF-β during the wound repair process.

### 5.2. CTHRC1 Is a Component of Canonical and Non-Canonical Wnt Signaling Pathways

CTHRC1 activity has also been linked to Wnt signaling. Canonical Wnt signaling is implicated in embryonic development, cancer, and stem cell differentiation [37,38]. Activation of the canonical Wnt signaling pathway leads to the stabilization and nuclear translocation of the transcriptional activator β-catenin into the cell nucleus, where β-catenin promotes the transcription of Wnt-associated genes [37]. Besides the canonical branch, there are several non-canonical branches of the pathway, which do not lead to the cytoplasmic stabilization of β-catenin. One of these pathways is the planar cell polarity (PCP) pathway, which regulates cell motility and adhesion via Dishevelled, RhoA, and actin cytoskeletal reorganization [37]. Canonical and noncanonical Wnt signaling pathways are also considered to be major modulators of RA pathogenesis [39].

**Figure 3:**
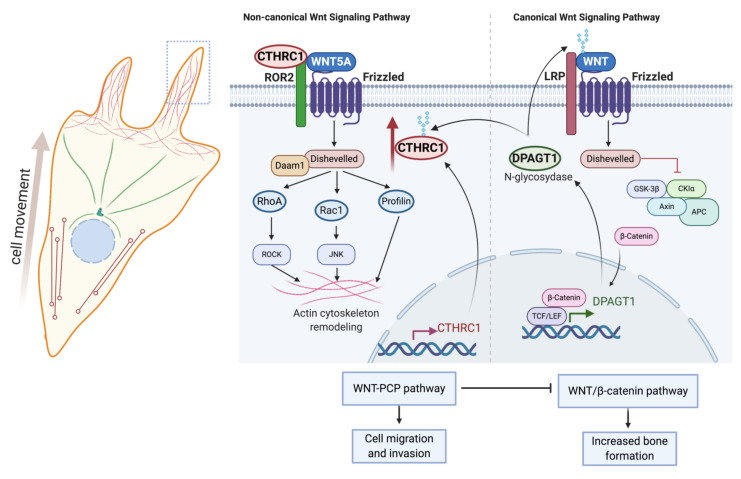
Role and regulation of CTHRC1 in canonical and noncanonical Wnt signaling. Expression of the N-glycosidase *DPAGT1* is induced in response to activation of canonical Wnt/β-catenin signaling. DPAGT1 mediates the N-glycosylation of Wnt ligands and of CTHRC1, thereby enhancing protein stability and secretion of CTHRC1. CTHRC1, in turn, attenuates signaling by the canonical Wnt/β-catenin pathway and induces cytoskeletal reorganization and cell movement via activation of noncanonical Wnt/PCP signaling. Secreted CTHRC1 activates the Wnt/PCP pathway by acting as a co-receptor and promoting the formation of the WNT5A/FZD/ROR2 complex. Created with BioRender.com.

Prevailing evidence suggests that canonical and non-canonical Wnt pathways converge at the level of CTHRC1. N-glycosylation by the Dolichyl-Phosphate N-Acetylglucosaminephosphotransferase 1 (DPAGT1) was reported to stabilize CTHRC1 protein stability and promote the secretion and pro-migratory function of CTHRC1 [40]. DPAGT1 expression is induced by activation of Wnt/β-catenin signaling [41], thus providing a link between Wnt/β-catenin and CTHRC1 (Figure 3) [40]. CTHRC1 may also positively promote the transcriptional activity of either β-catenin or the β-catenin/TCF complex [40], although it remains to be delineated exactly how CTHRC1 regulates Wnt/β-catenin signaling. Notably, in vitro studies showed upregulated expression of β-catenin in RA-FLS, leading to their chronic activation, indicating that Wnt signaling is induced in RA-FLS [42].

Various studies have shown that CTHRC1 can activate the Wnt/PCP signaling pathway by acting as a coreceptor for formation of WNT/FZD/ROR2 complexes [15]. Consistent with such a role, CTHRC1 has been reported to interact with several PCP core components, including multiple FZD (Frizzled) receptors (FZD3, FZD5 and FZD6), WNT5A and the non-canonical co-receptor ROR2 (Receptor Tyrosine Kinase Like Orphan Receptor 2), but not with LRP6 (Low-density lipoprotein receptor-related protein 6) and the PCP component VANGL2 (VANGL planar cell polarity protein 2) [15]. CTHRC1 promotes the binding of WNT5A to ROR2, leading to enhanced WNT5A-ROR2 complex formation and stimulation of RhoA and Rac1 small GTPase activation, indicating that CTHRC1 can modulate both cascades of the Wnt/PCP pathway (Figure 2) [15]. Furthermore, Wnt/PCP and CTHRC1 may be part of an autocrine feedback mechanism, because CTHRC1 gene expression has been reported to be induced upon activation of FZD6/WNT/PCP (Figure 2) [43].

A recent study further identified the vertebrate-specific transmembrane protein Wnt-activated inhibitory factor 1 (WAIF1, also known as trophoblast glycoprotein, TPBG; 5T4 oncofetal trophoblast glycoprotein) as a receptor for secreted CTHRC1. Wnt-Activated Inhibitory Factor 1 (WAIF1)/5T4 has been shown to inhibit Wnt/β-catenin signaling and to concomitantly activate noncanonical Wnt pathways [44]. Taken together, these data suggest that CTHRC1 expression and function are positively regulated by the canonical Wnt signaling pathway. CTHRC1, in turn, acts as a switch between canonical and noncanonical Wnt pathways, leading to the inhibition of the canonical branch and activation of the noncanonical WNT/PCP branch of the pathway (Figure 3).

## 6. CTHRC1 Is Associated with RA Development and Disease Severity

CTHRC1 was first linked to RA pathogenesis through the genetic association of *Cthrc1* gene polymorphisms with attenuation of proteoglycan-induced (PGIA) and collagen antibody-induced murine arthritis (CAIA) [45,46,47,48]. *Cthrc1* is located within the proteoglycan induced arthritis 8 (*Pgia8*) locus of mouse chromosome 15, which controls PGIA severity in a sex-specific manner [45,46,47,48]. In *Pgia8*-congenic male mice, expression of all genes located within the entire locus was suppressed by 30–50%, and this was linked to resistance to arthritis development [46]. However, of the over 200 genes located within this locus, *Cthrc1* expression exhibited the strongest correlation with arthritis severity and levels of the pro-inflammatory cytokines IL-6 and IL-1β [46].

Notably, while *Cthrc1* was the most significantly down-regulated gene in the locus, it also exhibited marked co-expression with several other genes located within the *Pgia8* locus: the Wnt signaling components R-spondyn (*Rspo2*) and Syndecan 2 (*Sdc2*), ADAM metallopeptidase with thrombospondin type 1 motif 12 (*Adamts12*), as well as Complement C1q and tumor necrosis factor related protein 3 (*C1qtnf3*) [46]. All these genes were significantly repressed in male mice and linked to inflammation. Co-expression of *Cthrc1* with *Rspo2* and *Sdc2* supports the notion that CTHRC1 and the Wnt signaling pathways are linked in RA. *Adamts12* is genetically associated with several inflammatory conditions, including asthma, Crohn’s disease, and RA [49,50]. In RA, ADAMTS12 is one of the enzymes triggering cartilage destruction by degrading cartilage oligomeric matrix protein (COMP) [46,51]. C1QTNF3 (also named CTRP3 for “C1q/TNF-related protein-3”) is an adipokine with broad immunomodulatory and metabolic functions [52]. *C1qtnf3/Ctrp3* is highly expressed in several mouse models of arthritis and attenuates systemic inflammation and arthritis severity, suggestive of a protective role [53,54].

Importantly, the syntenic region to the *Pgia8* locus of mouse chromosome 15 in the human genome is also associated with RA development, serum rheumatoid factor and efficacy of anti-TNF-α treatment of RA patients [55,56,57,58]. The linkage of CTHRC1 with arthritis development and disease severity in mice therefore raised the question of whether the corresponding human locus may be similarly linked to RA development in patients. Consistent with such a notion, we recently showed that CTHRC1 protein is significantly and specifically elevated in the plasma of RA patients [7]. Importantly, CTHRC1 plasma levels were low or undetectable in healthy controls, as well as in OA and ReA patients [7]. In addition, CTHRC1 levels were positively associated with RA disease markers, such as RF, anti-citrullinated protein antibodies (ACPA) and C-reactive protein (CRP). CTHRC1 also correlated significantly with RA disease activity based on the combined index of the 28-joint disease activity score (DAS28), the combined score DAS28-CRP, and with a panel of pro-inflammatory cytokines, including interleukin 1 beta (IL-1β), interleukin 6 (IL-6), interleukin 8 (IL-8) and interferon gamma (IFNγ) [7]. A recent study independently confirmed that CTHRC1 is a specific marker for the diagnosis of RA, particularly when used in combination with other markers, such as anti-mutated citrullinated vimentin antibodies (anti-MCV) [8]. Together, these findings corroborate observations in murine models of arthritis and indicate that CTHRC1 may have potential use as a biomarker for enhanced differential RA diagnosis. Moreover, CTHRC1 was also identified as a novel serum biomarker associated with disease activity in systemic lupus erythematosus (SLE, [59]). Interestingly, CTHRC1 protein serum levels were highest in a subgroup of SLE patients with arthritis [59]. Thus, CTHRC1 may serve as a marker for the development of arthritis in SLE.

## 7. Invasive Synoviocytes Are Key Drivers of Joint Destruction in RA

**Figure 4:**
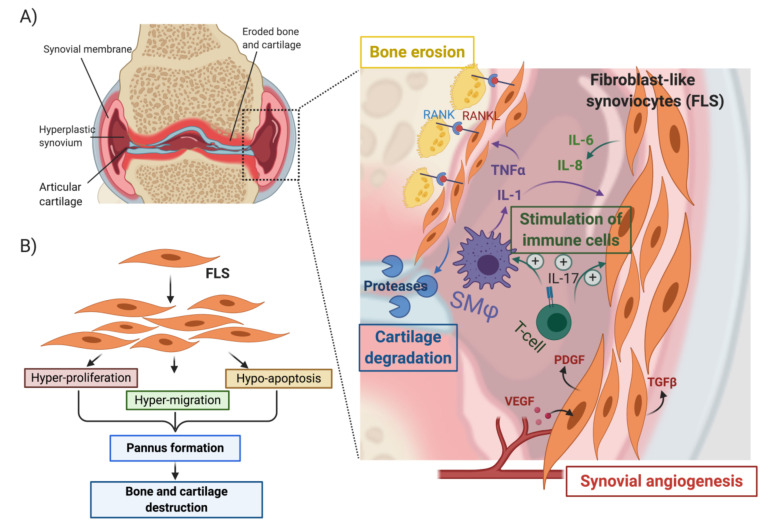
Role of synoviocytes in pannus formation and bone erosion in rheumatoid arthritis (**A**) and (**B**) activation of fibroblast-like synoviocytes by cytokines (TNF-α and IL-1β) secreted by synovial macrophages. (SMϕ) leads to the acquisition of a hyper-proliferative, hyper-migratory and hypo-apoptotic phenotype that contributes to the formation of a hyperplastic synovium and ultimately leads to bone and cartilage erosion. Activated synoviocytes in the pannus play a central role in the recruitment and stimulation of immune cells, the vascularization of the pannus through activation of angiogenesis, and the promotion of cartilage and bone erosion. Created with BioRender.com.

Synovial hyperplasia is a hallmark of RA pathogenesis and characterized by the formation of pannus. The arthritic pannus is a multicellular vascularized tissue composed of cells of both mesenchymal and hematopoietic origin [34,35]. In response to synovial inflammation, pannus tissue invades cartilage and bone, resulting in major damage of the intimal lining and sub lining layers of the synovial tissue and, eventually, results in joint destruction. Fibroblast-like synoviocytes (FLS) in the synovial intimal lining are key drivers of bone erosion in RA; these cells become hyperproliferative and acquire an aggressive migratory and invasive phenotype [60,61,62,63,64]. These cells are also a source of numerous pro-inflammatory cytokines, growth factors and cartilage- and bone-degrading proteases. (Figure 4A,B) [60,61,62,63,64]. RA-FLS are also known to co-operate with macrophage-like progenitor cells, leading to local formation of osteoclasts, which invade the subchondral bone using acid attack and acidic proteinases (Figure 4A). Pro-inflammatory cytokines like TNF-α and IL-1β further protect FLS from Fas-mediated cell death (Figure 4A), thus preventing the elimination of RA-FLS from the inflamed synovium [64,65]. RA-FLSs thereby establish an autocrine feedback network that perpetuates synovial hyperplasia, contributes to the inflammatory microenvironment and promotes pannus invasion through increased synoviocyte motility and invasion [64].

## 8. RA-FLS Are One Source of CTHRC1

Significantly, the immunohistochemical analysis of CAIA mouse synovium showed that CTHRC1 protein levels were highly elevated in pannus compared to normal synovial tissue and concentrated in FLSs located at the invasive leading edge of pannus [66,67,68]. Consistent with these murine studies, CTHRC1 was recently reported to be highly expressed in two synovium fibroblast subtypes isolated from tissues of RA patients. One FLS subtype, characterized by the presence of CD34 and cadherin 11 (CDH11) and the absence of THY1/CD90, was implicated in monocyte recruitment in the RA synovium through secretion of IL-6, CXCL12 (C-X-C Motif Chemokine Ligand 12) and CCL2 (C-C Motif Chemokine Ligand 2) [9,69]. A second fibroblast subtype (CD34^−^ THY1/CD90^+^ CDH11^+^) was significantly expanded in RA versus OA and exhibited the highest association with RA pathology [9,69]. Both *CTHRC1*-expressing FLS subtypes—the CD34^−^THY1^+^ and CD34^+^ subsets—also showed induced expression of several other genes related to migration and invasion, including *TWIST1*, *POSTN*, *LOXL2*, *PDGFRB* and *MMP14* [9,69]. Accordingly, both FLS subtypes exhibited enhanced migration and invasion in vitro. Thus, CTHRC1 may promote pathological changes in the synovium by modulating the migration and invasion of different FLS populations and, potentially, by contributing to the recruitment of immune cells to the inflamed synovium.

Nevertheless, at present, we can only speculate about the function of CTHRC1 in RA-FLS subpopulations and the mechanisms governing CTHRC1 expression in these cells. CTHRC1 might act as part of either Wnt or TGF-β signaling pathways. Canonical and noncanonical branches of the Wnt signaling pathway are considered to play major roles in RA pathogenesis, in part, by modulating the activation of FLS and by promoting the production of pro-inflammatory cytokines and chemokines [39]. Accordingly, upregulated expression of β-catenin and of non-canonical Wnt/PCP pathway members is observed in the RA synovium [70,71,72]. This is associated with chronic activation of RA-FLS and a pro-inflammatory microenvironment that promotes the local recruitment of immune cells [70,71,72]. Kim et al. demonstrated that WNT5A is a key inducer of cytokine production during inflammation [73]. *WNT5A* expression was observed in RA-FLS but not in normal tissue [74], and overexpression of *WNT5A* in RA-FLS led to enhanced production of pro-inflammatory cytokines and chemokines [72,73]. Conversely, blockade of non-canonical WNT5A/FZD5 signaling leads to downregulation of IL-6, IL-15 and RANKL, which inhibited RA-FLS activation [39,75]. These data indicate that WNT5A-mediated signaling induces the secretion of cytokines and chemokines that promote the recruitment of leukocytes into the synovium. The WNT5A/ROR2 complex also plays a crucial role in bone-marrow-derived mesenchymal stem cell differentiation into osteoblasts, suggesting that activation of non-canonical Wnt signaling in the arthritic synovium also affects bone homeostasis by modulating osteoblastogenesis [73]. Given that CTHRC1 is known to stabilize the Wnt/Frizzled complex [40], CTHRC1 could promote Wnt/PCP signaling in the synovium, thus potentially modulating inflammatory cell migration and cell differentiation, as well as bone remodeling.

While the importance of canonical and noncanonical Wnt signaling pathways in the arthritic synovium is well established, the role of TGF-β in the synovium and in RA disease pathogenesis is not well defined. TGF-β is known to modulate pathogenic and inflammatory responses in the RA synovium and to regulate osteoblast differentiation [76]. TGF-β1 may also promote synovial lining hyperplasia synergistically with TNF-α and IL-1β through the regulation of RA-FLS proliferation, invasion and migration (Figure 4) [77]. Future work will be important to characterize the functional roles of the unique fibroblast subsets of FLS marked by *CTHRC1* expression and to elucidate the specific mode of action and regulation of CTHRC1 in these FLSs.

## 9. CTHRC1 Plays a Central Role in Bone Remodeling

**Figure 5:**
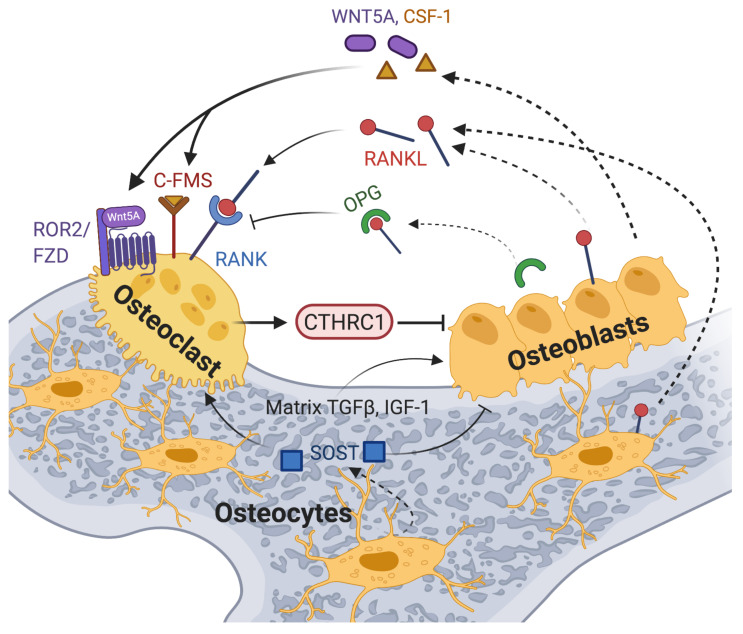
A model of CTHRC1 action in osteoclast–osteoblast crosstalk is shown, highlighting the effects of some of the key players involved. Membrane-bound and soluble RANKL produced by cells of the osteoblast lineage and by immune cells induces osteoclastogenesis upon binding to the receptor RANK on osteoclast precursors. RANKL action is opposed by the decoy receptor OPG secreted by osteoblasts or osteocytes. The inflammatory bone environment in RA results in increased production of RANKL by immune cells, osteoblastic cells and synovial fibroblasts. This exacerbates osteoclast differentiation and bone erosion. WNT5A binds to ROR2 receptors and activates non-canonical signaling, thereby promoting osteoclast differentiation and bone resorption. Likewise, signals generated by the binding of stromal-cell-produced colony-stimulating factor 1 (CSF-1) to the C-FMS receptor promote osteoclast differentiation and osteoclastogenesis. TGF-β and other factors, including IGF-1 released from the bone matrix during bone resorption, stimulate osteoblastogenesis [81]. CTHRC1 expression and secretion by osteoclasts points to an autoregulatory mechanism promoting osteoblastogenesis for enhanced bone formation [10,11,82,83], potentially by activating noncanonical Wnt signaling through the co-receptor WAIF1/5T4 [15,83]. An alternative model proposed by Jin et al. implicates osteoblast- and osteocyte-secreted CTHRC1 in the negative regulation of osteoclast differentiation through inhibition of RANKL-RANK signaling [84]. Created with BioRender.com.

Bone erosion is a central aspect of RA and is associated with disease severity [78]. Bone lesions both within and around the affected joints often appear early in the disease and can be accompanied by widespread osteoporosis in some patients without effective treatment. These lesions are the result of deregulated bone homeostasis due to enhanced osteoclast differentiation associated with bone resorption and the inhibition of osteoblast-mediated bone formation [78]. Osteoclasts are multinucleated cells that differentiate from monocyte/macrophage precursors under osteoblast/osteocyte control [78]. Osteoblasts regulate osteoclast differentiation and function via colony stimulating factor-1 (CSF-1) and RANKL (Receptor activator of nuclear factor κB/Receptor activator of nuclear factor kappa-Β ligand, Figure 5) [78]. RANKL, which binds to the receptor RANK expressed on the surface of osteoclast precursors [79], is also secreted by osteocytes (Figure 5). In addition, osteoclast differentiation through RANK-RANKL interactions can be initiated by RA-FLSs, which also express RANKL on their surface [80].

Osteoblasts and osteocytes also balance osteoblast and osteoclast differentiation through secretion of additional factors. Osteoblasts secrete osteoprotegerin (OPG), a soluble receptor for RANKL, which inhibits osteoclastogenesis by blocking the RANKL-RANK interaction, and WNT5A, which contributes to the regulation of osteoclastogenesis through activation of noncanonical Wnt signaling (Figure 5) [85]. Studies showed that RANKL/RANK-deficient mice develop osteopetrosis, whereas OPG-deficient mice develop osteoporosis of trabecular and cortical bone [48,49,50,51]. Osteocytes also produce sclerostin (SOST), a secreted protein that attenuates osteoblast differentiation and promotes osteoclastogenesis by inhibiting the Wnt/β-catenin pathway [86]. While osteoclast-mediated bone resorption and osteoblast-mediated bone formation are normally tightly coupled to maintain bone homeostasis, the inflammatory bone environment in RA results in increased production of RANKL by immune cells, osteoblastic cells and synovial fibroblasts [78,87]. This shifts the balance towards pathological osteoclastogenesis and bone resorption.

Importantly, CTHRC1 has emerged as a critical coupling factor that links bone resorption to bone formation by controlling osteoblast-osteoclast cross-talk [10,11,82,83]. The essential regulatory role of CTHRC1 in bone homeostasis has been clearly demonstrated in vivo in mouse studies. Accordingly, loss of CTHRC1 function in mice was shown to result in decreased bone mass and decreased bone formation due to impaired coupling processes [10,11,84]. Conversely, overexpression of *Cthrc1* increased bone mass through stimulation of bone formation in transgenic animals [10,11,84].

However, the cellular source of secreted CTHRC1 and its precise role in bone biology are currently somewhat controversial, and any role of CTHRC1 in bone erosion in RA patients remains to be defined. While CTHRC1 may modulate osteoclastogenesis in the synovium via its expression in specific RA-FLS subsets [9,69], CTHRC1 clearly also plays an independent role in the regulation of bone formation/resorption. Several studies reported that CTHRC1 is secreted from osteoclasts and stimulates osteoblast differentiation (Figure 5 and Table 1) [10,11,82,83]. This effect may in part be mediated through WAIF1/5T4, a regulator of Wnt/β-catenin signaling expressed on stromal cells [15,83]. WAIF1/5T4 has been proposed to act as a receptor for osteoclast-derived CTHRC1, leading to the formation of a ternary complex by CTHRC1, WAIF1 and ROR2 on the osteoblast surface, which then mediates osteoblast-osteoclast crosstalk for bone remodeling [15,83]. Consistent with the notion that CTHRC1 is produced by osteoclasts to influence bone formation, the cell-type-specific ablation of *Cthrc1* expression in murine osteoblasts had no significant effect on bone mass, whereas osteoclast-specific deletion of *Cthrc1* led to bone loss [11]. Based on these data, Takeshita and coworkers concluded that CTHRC1 is secreted by osteoclasts and plays a major role in bone formation [11].

In contrast to the aforementioned studies, Jin et al. reported that murine CTHRC1 is produced by osteocytes and osteoblasts, but not osteoclasts. Accordingly, *Cthrc1* was highly expressed by osteoblasts lining the trabecular and cortical bone surface and by some osteocytes but was absent from multinucleated osteoclasts [84]. Loss of *Cthrc1* expression in *Cthrc1* null mice was associated with a significant decrease in trabecular bone mass due to upregulated bone resorption, whereas cortical bone formation was only minimally affected [84]. Notably, bone-marrow-derived monocytes isolated from *Cthrc1* null mice showed normal osteoclast differentiation in vitro [84]. However, osteoclastogenesis could be blocked by the addition of recombinant CTHRC1 to these cells, indicating that CTHRC1 acts exogenously. Overall, these results led the authors to conclude that osteoblast- and osteocyte-derived CTHRC1 plays a critical role in negatively regulating osteoclast differentiation (Table 1, [84]). CTHRC1 may block osteoclastogenesis through suppression of RANKL expression and by inhibiting RANKL-induced NFκB activation (Table 1, [84]).

Overall, the molecular and cellular basis for the discrepancy between data reported from different laboratories remains to be defined. Contributing factors may be the use of different Cre-lox recombination approaches to generate cell-specific gene inactivation of *Cthrc1* in mice and the distinct detection methods used to visualize CTHRC1 protein. Alternatively, CTHRC1 may affect bone homeostasis through a combination of mechanisms and pathways depending on the tissue context and the presence or absence of inflammatory conditions. Additional studies in cellular and in vivo systems will be required to resolve the discrepancy between the data reported by different investigators and to clarify the precise physiological activity CTHRC1 in bone homeostasis. Regardless of this discrepancy, the role of CTHRC1 in bone remodeling has been firmly established in animal models and this may also have implications for the development of cartilage and bone erosion in RA patients.

Significantly, recent studies in mice focused on a potential role of CTHRC1 in joint destruction. Jin et al. assessed the role of murine CTHRC1 in arthritis development by studying the effect of *Cthrc1* ablation on collagen antibody-induced arthritis [84]. Interestingly, *Cthrc1* null mice exhibited exacerbated arthritis with extensive inflammatory cell infiltration and pannus formation and significant bone erosion. Thus, CTHRC1 appears to confer potent anti-inflammatory effects in the synovium and may play an important and broader role in the regulation of the immune response in this mouse model of arthritis. It remains to be seen whether these effects also translate to RA patients and whether elevated levels of CTHRC1 confer anti-inflammatory effects and act to reduce arthritic joint destruction.

## 10. Sex Disparity and CTHRC1

Rheumatoid arthritis occurs more frequently in women (about 75%), and symptoms are more pronounced in this population [88]. In particular, the influence of sex hormones on the immune response is well established [89], and estrogens are one of the causes of female predominance in RA and highly linked to disease severity and effects on bone remodeling [90]. In this regard, it is interesting to note that the murine *pgia8* locus conferred sex-specific attenuation of arthritis only in male, but not female mice [46]. Furthermore, the expression of *Cthrc1*, metalloproteinase *Adamts12*, *Rspo2* and *Sdc2* genes was not only highly associated with disease severity but was also linked to the sex-specific effects conferred by the *pgia8* locus on arthritis resistance [46]. The attenuation of *Cthrc1* expression in male mice was linked to the *pgia8* locus because CTHRC1 mRNA levels were equal in wild-type male and female mice [46]. The molecular basis for these sex-specific effects is not known. However, recent studies reported by Jin et al. show that CTHRC1 also has a diverse effect on bone formation in male and female mice. Bone histomorphometry, micro-computed tomography analysis and functional readouts of bone strength showed bone formation impairment of trabecular and cortical bone in male *Cthrc1* null mice with a significant reduction in bone mass, whereas female *Cthrc1* null mice exhibited impairment only in trabecular bone [84]. Taken together, these results indicate that in mice the sex-specific disparities in RA are linked to transcriptional regulation of *Cthrc1* and genes involved in cartilage degradation (*Adamts12*) and canonical and noncanonical Wnt signalling (*Rspo2*, *Sdc2*). Likewise, CTHRC1 confers sex-specific effects to bone formation in mice. Whether *CTHRC1* expression or function is also linked to the sex-bias phenotype of RA in human patients remains to be shown.

## 11. Conclusions

In this review, we described recent advances examining the role of CTHRC1 in RA pathogenesis. Available data suggest that CTHRC1 represents a promising new diagnostic and, potentially, also prognostic biomarker of RA. Additional studies will be necessary to reveal the precise function of CTHRC1 in RA pathogenesis in patients, such as the role of CTHRC1 in the RA synovium and the development of bone erosion. Additional studies will also be required to further address the mechanisms controlling CTHRC1 expression and function. In particular, the possibility that CTHRC1 signaling has different functional effects in specific cell types, where it may modulate TGF-β and/or Wnt signals depending on cellular context. Nevertheless, the similarities in CTHRC1 expression and function in rodent models of arthritis and RA patients are intriguing and provide a basis for future studies exploring the therapeutic and diagnostic potential of CTHRC1.

## Figures and Tables

**Table 1:** Potential cellular sources of CTHRC1 and effects within the joint.

Cell Type	Potential Effect on Cells	Reference
Rheumatoid arthritis fibroblast-like synoviocytes (RA-FLS)	Proliferation, migration, initiation of cartilage destruction	[66]
Osteoclasts	Inhibition of osteoblast differentiation and osteoblast driven bone formation;	[10,11,82,83]
	activates WAIF1/PKCδ/ERK pathway necessary for RANKL expression leading to reduced bone resorption and formation	[83]
Osteoblasts	Inhibition of monocyte-osteoclast differentiation and osteoclast-driven bone resorption in trabecular bone, inhibition of NFκB-dependent signaling; CTHRC1 may additionally suppress RANKL expression	[84]
Osteocytes	Inhibition of monocyte-osteoclast differentiation and osteoclast driven bone resorption in trabecular bone, inhibition of NFκB-dependent signaling; CTHRC1 may additionally suppress RANKL expression	[84]

## Abbreviations

ACPA	Anti-citrullinated protein antibody
ADAMTs12	A disintegrin and metallopeptidase with thrombospondin type 1 motif 12
Ang2	angiopoietin 2
BMP2/4	Bone morphogenetic protein 2/4
CAIA	Collagen Antibody-Induced Arthritis
CCL2	C-C Motif Chemokine Ligand 2
CD34, CD90	Cluster of differentiation 34/90
CDH11	cadherin 11
COMP	cartilage oligomeric matrix protein
C1qtnf3	Complement C1q tumor necrosis factor-related protein 3
CRP	C-reactive protein
CSF-1	Colony stimulating factor 1
CTHRC1	Collagen triple helix repeat-containing 1 protein
CXCL12	C-X-C Motif Chemokine Ligand 12
Daam2	Dishevelled associated activator of morphogenesis
DPAGT 1	Dolichyl-Phosphate N-Acetylglucosaminephosphotransferase 1
Dvl	Dishevelled
FZD	Frizzled
GSK3β	Glycogen synthase kinase 3beta
IL-1/6/8/11/15	Interleukin 1/6/8/11/15
INF-γ	Interferon gamma
LRP	Lipoprotein receptor-related protein
OA	Osteoarthritis
OPG	Osteoprotegerin
PCP Pathway	Planar cell polarity pathway
Pgia8	Proteoglycan induced arthritis 8
RA	Rheumatoid arthritis
RA-FLS	Rheumatoid arthritis fibroblast like synoviocyte
RANKL	Receptor Activator of Nuclear Factor-Kappa B ligand
RF	Rheumatoid factor
ROR2	Receptor tyrosine kinase-like orphan receptor 2
RSPO2	R-spondin 2
Sdc2	Syndecan 2
SLE	Systemic lupus erythematosus
SMAD 2/3/4	The abbreviation refers to the homologies to the *Caenorhabditis elegans* “small” worm phenotype and Drosophila MAD (“Mothers Against Decapentaplegic”) family of genes
SOST	Sclerostin
TPBG	trophoblast glycoprotein
TCF	T cell factor
TGF-β	Transforming growth factor beta
TNF-α	Tumor necrosis factor alpha
THY1	Thy-1 Cell Surface Antigen
VANGL2	VANGL planar cell polarity protein 2
WAIF1	Wnt-activated inhibitory factor 1
Wnt	Wingless and Int-1
